# Nutrient Exchange of Carbon and Nitrogen Promotes the Formation of Stable Mutualisms Between *Chlorella sorokiniana* and *Saccharomyces cerevisiae* Under Engineered Synthetic Growth Conditions

**DOI:** 10.3389/fmicb.2019.00609

**Published:** 2019-03-26

**Authors:** René K. Naidoo, Zoë F. Simpson, Jennifer R. Oosthuizen, Florian F. Bauer

**Affiliations:** Department of Viticulture and Oenology, Institute for Wine Biotechnology, Stellenbosch University, Stellenbosch, South Africa

**Keywords:** microalgae, yeast, synthetic, engineered, obligatory, mutualism

## Abstract

Microbial biotechnological processes can be based on single species pure cultures or on multi-species assemblages. While these assemblages can be advantageous by offering more functionalities and more resilience to changing environmental conditions, they can be unpredictable and difficult to control under synthetically engineered growth conditions. To overcome the unpredictable nature of these microbial assemblages, the generation of stable mutualistic systems through synthetic ecology approaches may provide novel solutions for understanding microbial interactions in these environments. Here we establish a stable association between two evolutionarily unrelated, but biotechnologically complementary species isolated from winery wastewater; a strain of the yeast *Saccharomyces cerevisiae* and microalga, *Chlorella sorokiniana*. Yeast and microalgae were able to form obligate (interdependent) and non-obligate (facultative) mutualisms under engineered batch co-culture growth conditions. Obligate mutualism was maintained through the reciprocal exchange of carbon and nitrogen where the yeast ferments mannose to produce carbon dioxide for use by the microalga; and the microalga provides the yeast with nitrogen by metabolizing nitrite to ammonium. The effect of temperature and pH on the establishment of these mutualisms was evaluated and pH was found to be a key determinant for mutualism formation under obligatory conditions. Moreover, the combinations of the two species under non-obligatory growth conditions led to improvement in growth rate and biomass production when compared to single species cultures grown under the same conditions. Such engineered mutualisms are the first step in developing stable multi-species assemblages, while providing a system to generate novel insight into the evolution of mutualistic interactions between phylogenetically distant microorganisms.

## Introduction

Photosynthetic microalgae are considered the most efficient producers of biomass for the production of biofuels (bioethanol, biohydrogen, biodiesel, and biogas) and bio-products (animal feeds, omega fatty acids, and carotenoids) (John et al., 2011; Jones and Mayfield, 2012; Borowitzka and Moheimani, 2013). However, most microalgae-based processes have not delivered as expected due to a lack of cost-effective technologies for the production of algal biomass at an industrial scale. This is due in part to the use of single species, monoculture systems, which work well in laboratory settings but present numerous challenges when up-scaled. These systems are often unstable, prone to contamination and, as a consequence, show inconsistent biomass production (Cai et al., 2013). Moreover, maintaining sterility in large facilities can be a costly and labor intensive process (Kazamia et al., 2012).

Synthetic microbial ecology offers a solution to this problem as multi-species assemblages comprised of microorganisms with complementary metabolic capabilities are generally more robust in the face of environmental change than their monoculture counterparts (Brenner et al., 2008). These artificial assemblages can be designed to incorporate key design elements such as species specific selection and engineered symbiosis; which can serve to improve processes such as wastewater treatment and biofuel production (Lee, 2001; Kazamia et al., 2014). Synthetic microbial ecology has been previously defined as “the rational and theory driven manipulation of artificial ecosystems” (Kazamia et al., 2012; De Roy et al., 2014; Dolinšek et al., 2016; Stenuit and Agathos, 2016). This definition has been further extended to incorporate the use of engineering principles in the design, construction and quantitative analysis of artificial microbial ecosystems (Kazamia et al., 2012; de-Bashan et al., 2016; Dolinšek et al., 2016). Thus, synthetic ecology seeks to understand how microbial interactions influence community structure under carefully controlled experimental conditions (Dolinšek et al., 2016). The benefit of such an approach is that it reduces the complexity often observed within natural ecosystems and makes these systems more amenable to mathematical modeling. This allows one to predict the behavior of organisms in microbial consortia in response to changing environmental conditions (Dolinšek et al., 2016). Additionally, these artificial microbial communities can often perform more complex tasks and the network of interactions between microorganisms results in more stable and robust ecosystems (Brenner et al., 2008; de-Bashan et al., 2016).

The mutualistic interactions between microalgae and fungi/yeast have been harnessed effectively to improve the bioremediation efficiency in several biological wastewater treatment studies (Cheirsilp et al., 2011; Wrede et al., 2014; Muradov et al., 2015). Specifically, the use of microalgae and oleaginous yeasts in an integrated co-culture system for wastewater treatment resulted in enhanced lipid production (Cheirsilp et al., 2011; Papone et al., 2015). While the benefits that can be derived from these systems are clear, the nature of mutualistic interactions between yeast and microalgae in co-culture systems are still largely unexplored.

Hom and Murray (2014) demonstrated that obligate mutualisms between the yeast *Saccharomyces cerevisiae* and the microalga *Chlamydomonas reinhardtii* were relatively easy to establish under strongly selective conditions. They also demonstrated that these mutualisms were phylogenetically broad as they were established with four *Chlamydomonas* species and many different ascomycetous yeasts spanning 300 million years of evolutionary divergence in each clade (Hom and Murray, 2014). These photoautotroph-heterotroph partnerships have also been shown to afford some advantages to processes such as biofuel production. High oxygen accumulation, especially in closed systems, poses a significant problem for algal growth as it inhibits photosynthesis. However, the inclusion of an oxygen consuming heterotrophic partner would mitigate this problem while resulting in increased yeast biomass and metabolite production (Ota et al., 2011; Hays et al., 2017; Li et al., 2017). Furthermore, these engineered symbiotic cultures may be a novel means of assembling communities with a diverse range of functional and metabolic capabilities (de-Bashan et al., 2016; Dolinšek et al., 2016).

Winery wastewater environments are populated by a diverse array of microorganisms which are constantly exposed to flunctuating enviromental conditions. This is due mainly to to the variable composition of the wastewater which is a consequence of seasonal harvesting and wine production processes (i.e., vintage, racking, and bottling) (Ioannou et al., 2015). Winery wastewaters also often have disproportionate carbon:nitrogen:phosphorus ratios and high organic loads predominated mainly by sugars, organic acids, alcohols, esters, and polyphenols (Rodríguez et al., 2007; Strong and Burgess, 2008; Mosse et al., 2011a,b). Consequently, robust biological treatment systems which are able to buffer changes in salt, pH, temperature and organic load are required when developing biological winery wastewater treatment processes (Ioannou et al., 2015). Unfortunately, appropriate strain selection which is a key requirement for the development of these processes has not received much attention. We suggest that multi-species assemblages comprising yeast and microalgae in mutualistic associations might offer increased stability and improved bioremediation efficiency under these variable conditions.Yeasts are attractive candidates for winery wastewater bioremediation as they are readily able to break down the sugars and organic acids which dominate these environments and serve to decrease the chemical oxygen demand (COD) in numerous wastewasters (Malandra et al., 2003; Gharsallah, 2008; Bezuneh, 2016; Wang et al., 2018). These heterotroph-photoautotroph partnerships could also serve to enhance the efficiency of integrated aerobic wastewater treatment processes, with each partner producing either O_2_ or CO_2_ for use in valuable biomass production. Additionally, the reduction of toxic reactive oxygen species by the heterotroph partner has been shown to protect the phototroph from oxidative stress in these co-cultures sytems (Li et al., 2017). Winery wastewater environments are usually characterized by low nitrogen levels with the most commonly detected forms of nitrogen in winery wastewater being ammonium, nitrate, and nitrite which can be easily assimilated by microalgae (Kalyuzhnyi et al., 2001; Glass et al., 2009; Casazza et al., 2016; Liu et al., 2016; Welz et al., 2016;Chen et al., 2017; Zhang et al., 2017; Higgins et al., 2018). Thus, the ability of most microalgae to assimilate a wide variety of nitrogen sources would be advantageous in these low nitrogen winery wastewater environments where they can alternate between different nitrogen sources (Glass et al., 2009; Chen et al., 2017). Moreover, controlling the carbon and nitrogen flux in these nitrogen limited environments through co-culture interaction, may induce lipid accumulation in various *Chlorella* species as nitrogen limitation has been shown to increase lipid yields (Rodolfi et al., 2009; Zhang et al., 2013; Griffiths et al., 2014; Chen et al., 2017).

In this study, as a first step to building a functional multi-species wastewater ecosystem we aim to gain a better understanding of the nature of the interactions between yeast and microalgae by establishing stable obligate mutualisms between naturally occurring winery wastewater yeast and microalgae. These mutualisms were established under strongly selective conditions, based on the reciprocal exchange of carbon and nitrogen, in growth media free of any contaminating microorganisms. The impact of temperature and pH, two key variables in winery wastewater, on single and co-cultures was evaluated prior to the optimization of batch bioreactor conditions. Furthermore, combinations of the species under non-obligatory mutualistic growth conditions led to improvement in growth and biomass production under model experimental conditions compared to single species cultures. This study provides evidence for the benefits that can be derived from co-culture systems under non-optimal growth conditions and highlights the potential of synthetic ecological approaches as well as the ease with which these yeast and microalgae obligate mutualisms can be established.

## Materials and Methods

### Sampling, Isolation, and Identification of Microorganisms

Winery wastewater was sampled from a winery in the Stellenbosch wine region (Stellenbosch, South Africa) in November 2014. This winery wastewater was brownish in color with a COD of 4140 mg/ml and a pH of 4. Microalgae and yeasts were cultured from wastewater samples and selective media was used for the isolation of groups of microorganisms. This included Dichloran Rose Bengal agar (DCRB, Sigma) to select for yeasts and Bold Basal medium with three-fold nitrogen (3N BBM-V) a nutrient rich medium, to select for microalgae (Bold, 1949). Microalgae isolates were transferred to fresh 3N BBM-V agar plates, followed by streaking for single colonies. Microalgae are currently maintained in liquid medium and preserved as algae slope cultures. Yeasts which differed visually were transferred to fresh agar plates, streaked to obtain pure cultures and glycerol stocks of each isolate were stored at -80°C.

### Genomic DNA Extraction, PCR, and Sequencing

Genomic DNA was extracted from each isolate according to Hoffman and Winston (1987) and used as a template for 18S rRNA (Algae) and ITS-5.8S (Yeast) PCR reactions (Hoffman and Winston, 1987). PCR reactions (50 μl) contained 50 ng of yeast genomic DNA (KW 4 or KW 13), 0.5 μM ITS1 primer (5′TCCGTAGGTGAACCTGCGG 3′), 0.5 μM ITS4 primer (5′ TCCTCCGCTTATTGATATGC 3′),1 × buffer (10 × colorless ExTaq Buffer^^®^^), 0.25 mM dNTPs, 2 mM MgCl_2_, and 1.25 U Ex Taq [Promega^^®^^, (Ghosh et al., 2015)]. PCR reactions for microalgae isolates were as described for the yeast isolates, however, 0.5 μM 18S rRNA primers (5′ ACCTGGTTGTCCTGCCAGT 3′ and 5′ TCAGCCTTGCGACCATAC 3′) were used instead of the ITS primers (Pereira et al., 2013). PCR cycling conditions were as follows: 3 min at 94°C, 30 s at 94°C, 54°C for 30 s, 72°C for 45 s for 30 cycles and a final elongation step for 10 min at 72°C. The PCR products were separated by gel electrophoresis in a 0.8% gel and were excised and purified with the Zymoclean^TM^ Gel DNA Recovery Kit. Sequencing was performed, using the primers listed above, on an ABI Prism 377 automated DNA sequencer at the Central Analytical Facility at Stellenbosch University. ITS-5.8S rRNA and 18S rRNA gene sequences were assembled using DNAMAN analysis software version 4.15 (Lynnon BioSoft). The nucleotide sequences obtained from each of the isolates were compared using the BLAST (Basic local alignment search tool) algorithm with the available sequences in GeneBank at National Center for Biotechnology Information (NCBI) (Altschul et al., 1997).

### Selection of Carbon and Nitrogen Sources

To develop conditions which promote the formation of obligate mutualisms between yeast and microalgae in this study, the identification of a carbon source which the microalgae cannot use for growth, was a key requirement. The ability of *Chlorella sorokiniana* to utilize glucose, fructose, mannose, galactose, ethanol, and glycerol as carbon sources and *S. cerevisiae* to use nitrite were evaluated over a period of 7 days. These experiments were conducted under mono- and co-culture conditions in 10 ml volumes in airtight culture tubes containing tris-acetate-phosphate (TAP) medium + 3.6% carbon source + 20 mM potassium nitrite (KNO_2_) + 1X Hom’s vitamins/liter (4 mg myo-inositol, 0.4 mg 4-amino benzoic acid, 0.4 mg calcium D-pantothenate, 0.4 mg niacin, 0.4 mg pyridoxine hydrochloride, 0.4 mg thiamine hydrochloride, 0.002 mg biotin, 0.002 mg cyanocobalamin, 0.002 mg folic acid, pH, 6.8) pers. communication, Erik F. Y. Hom at 25°C with continuous illumination (46 μmol m^-2^ s^-1^) and a starting pH of 7 (Table 1). Samples were taken at day 0 and 7 and cell counts performed to determine increases in cell density.

**Table 1:** Monoculture, co-culture, and bioreactor batch culture conditions for *S. cerevisiae* and *C. sorokiniana*.

Growth condition	Carbon source	Nitrogen source	Microorganisms	Temperature (°C)	pH
Monoculture	Mannose	Ammonium	*S. cerevisiae*	25, 30, and 37	5, 6, 7, 8, and 9
Monoculture	Glucose	Nitrite	*C. sorokiniana*	25, 30, and 37	5, 6, 7, 8, and 9
Monoculture	Glycerol	Nitrite	*S. cerevisiae*	25	7
Monoculture	Ethanol	Nitrite	*S. cerevisiae*	25	7
Monoculture	Maltose	Nitrite	*S. cerevisiae*	25	7
Monoculture	Mannose	Nitrite	*S. cerevisiae*	25	7
Monoculture	Fructose	Nitrite	*S. cerevisiae*	25	7
Monoculture	Sucrose	Nitrite	*S. cerevisiae*	25	7
Monoculture	Galactose	Nitrite	*S. cerevisiae*	25	7
Monoculture	Glucose	Nitrite	*S. cerevisiae*	25	7
Monoculture	Glycerol	Nitrite	*C. sorokiniana*	25	7
Monoculture	Ethanol	Nitrite	*C. sorokiniana*	25	7
Monoculture	Maltose	Nitrite	*C. sorokiniana*	25	7
Monoculture	Mannose	Nitrite	*C. sorokiniana*	25	7
Monoculture	Fructose	Nitrite	*C. sorokiniana*	25	7
Monoculture	Sucrose	Nitrite	*C. sorokiniana*	25	7
Monoculture	Galactose	Nitrite	*C. sorokiniana*	25	7
Monoculture	Glucose	Nitrite	*C. sorokiniana*	25	7
***Non-obligate mutualisms***					
Co-culture	Glycerol	Nitrite	*S. cerevisiae + C. sorokiniana*	25	7
Co-culture	Ethanol	Nitrite	*S. cerevisiae + C. sorokiniana*	25	7
Co-culture	Maltose	Nitrite	*S. cerevisiae + C. sorokiniana*	25	7
Co-culture	Fructose	Nitrite	*S. cerevisiae + C. sorokiniana*	25	7
Co-culture	Sucrose	Nitrite	*S. cerevisiae + C. sorokiniana*	25	7
Co-culture	Galactose	Nitrite	*S. cerevisiae + C. sorokiniana*	25	7
Co-culture	Glucose	Nitrite	*S. cerevisiae + C. sorokiniana*	25	7
Monoculture	Acetic acid + Mannose	Nitrite	*S. cerevisiae*	25	7
Monoculture	Acetic acid + Mannose	Nitrite	*C. sorokiniana*	25	7
Co-culture	Acetic acid +Mannose	Nitrite	*S. cerevisiae + C. sorokiniana*	25	7
Monoculture	Acetic acid + Mannose	Ammonium	*S. cerevisiae*	25	7
Monoculture	Acetic acid + Mannose	Ammonium	*C. sorokiniana*	25	7
Co-culture	Acetic acid + Mannose	Ammonium	*S. cerevisiae + C. sorokiniana*	25	7
***Obligate mutualisms***					
Co-culture	Mannose	Nitrite	*S. cerevisiae + C. sorokiniana*	25, 30, and 37	5, 6, 7, 8, and 9
Batch bioreactor	Mannose	Nitrite	*S. cerevisiae + C. sorokiniana*	25	8

### Pre-culture Growth Conditions

A methodology similar to that described by Hom and Murray (2014) was employed with some modifications. *S. cerevisiae* and *C. sorokiniana* were cultured for 16 h to log phase in 5 ml TAP medium + 2% mannose + 1X Hom’s vitamins/litre. The antibiotic chloramphenicol was added to all pre-cultures at a concentration of 340 μg/ml to inhibit the growth of bacteria. Yeast and microalgae pre-cultures were centrifuged at 2000 × g (alga) or 4000 × g (yeast) for 3 min to pellet cells. Cells were re-suspended in a volume of TAP media without nutrients equivalent to the pre-culture volume. The cells were washed two more times, with the final volume being half of the initial volume. Cell densities were determined by doing microscope counts using a haemocytometer and a stock concentration of each cell type was prepared in fresh TAP medium lacking nutrients for single or co-culture inoculation.

### Experimental Growth Conditions

**Figure 1:**
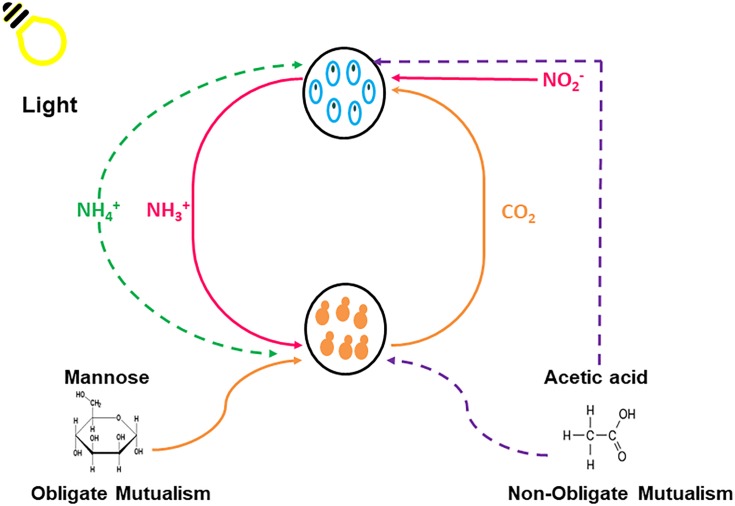
Graphic illustration of the experimental design for the obligatory and non-obligatory experiments showing the various nutrients that are exchanged between *Chlorella sorokiniana* (Blue) and *Saccharomyces cerevisiae* (Orange) within the synthetically engineered environment. Solid lines represent obligate conditions and dashed lines represent non-obligate conditions.

All growth experiments were conducted in TAP medium, lacking a carbon (C) and nitrogen (N) source, supplemented with 1X Hom’s vitamins. The experimental scheme for these experiments are displayed in Figure 1 and Table 1. The obligatory growth conditions developed here create an environment wherein each microorganism is reliant on its partner for growth and survival. Non-obligate growth refers to a condition where one species could survive without the other species, but it is better for both species to maintain the relationship.

### Obligatory Conditions

Mannose and nitrite were used to induce obligate mutualisms between *S. cerevisiae* and *C. sorokiniana* facilitated by the reciprocal exchange of C and N (Table 1). Mannose was added to a final concentration of 3.6% and nitrite to a final concentration of 20 mM. *S. cerevisiae*; and *C. sorokiniana* cells were inoculated to a starting cell density of 0.1 × 10^6^ cells/ml. Co-culture experiments were conducted at 25, 30, and 37°C without agitation with continuous light (46 μmol m^-2^ s^-1^, Sper Advanced Light Meter 840022) and cell densities were determined at the time-points mentioned below. Co-culture growth was also assessed in TAP media with a starting pH of between 5 and 9 at a temperature of 25°C. Three replicate experiments were performed and at appropriate experimental time-points (Day 0, 3 5, 7, and 11) cultures were thoroughly mixed by vortexing and the cell densities were measured using microscopic haemocytometer cell counts to evaluate mutualistic behavior. For each experiment 3 biological replicates were counted, and three separate co-cultures were sacrificed for each experimental measurement at each time point. Cultures were regularly monitored for contamination using microscopy, with periodic plating out of yeast and algae cultures on yeast peptone dextrose (YPD) agar and TAP agar.

### Non-obligatory Conditions

Tris-acetate-phosphate medium supplemented with 1X Hom’s vitamins, 0.1 % acetic acid, 3.6% mannose and 20 mM KNO_2_ was used for single and co-culture growth under non-obligate conditions (Table 1). Similarly, TAP medium supplemented with 1X Hom’s vitamins, 0.1 % acetic acid, 3.6% mannose, and 20 mM NH_4_Cl was used for single and co-culture growth experiments. Growth was evaluated at appropriate time points at a temperature of 25°C and a pH of 7. Cells were inoculated to a density of 0.1 × 10^6^ cells/ml. Experimental measurements were made as described above.

### Obligate Mutualistic Growth in 1L Bioreactor

Starter cultures were prepared with a methodology similar to that described previously. Cells were co-cultured in modified TAP medium (pH 8), which consisted of 500 ml TAP medium lacking ammonium (NH_4_Cl), supplemented with 3.6% mannose, 20 mM KNO_2_ and 1X Hom’s vitamins (Table 1). Cells were inoculated to a cell density of 1 × 10^6^ cells/ml. Co-culture experiments were conducted at 25°C, with 50 rpm agitation under continuous light (∼200 μmol m^-2^ s^-1^) in a BioFlo 110 Vessel bioreactor system (New Brunswick Scientific) and the pH, dissolved oxygen concentration, agitation and temperature were monitored throughout the course of the experiment. The batch bioreactor system was set-up as described in the Guide to Operations manual no. M1273-0054 (Figure 4, New Brunswick Scientific). At appropriate experimental time-points (12-h interval during the day), co-cultures were thoroughly mixed by increasing agitation to 300 rpm for 5 min, thereafter the agitation was set back to 50 rpm. Five ml were sampled from the bioreactor every 12 h for 7 days. Yeast and microalgae growth was monitored by means of haemocytometer cell counts (cell/ml) and combined dry weight (g/l). Mannose (D-Mannose/D-Fructose/D-Glucose Assay kit, Megazyme) and nitrite (Nitrite/Nitrate Assay Kit, colorimetric, Merck) consumption were monitored throughout the experiment. The production of organic acids (citric acid, tartaric acid, malic acid, succinic acid, and acetic acid), glycerol and ethanol were measured using high-performance liquid chromatography (HPLC) analysis (Eyéghé-Bickong et al., 2011). The HPLC method (SUG_PY5) was run using an isocratic gradient of 5 mM H_2_SO_4_ at a flow rate of 0.5 ml/min for 39 min, and the injection volume was 10 μl. After HPLC analysis, the integrated standard area and known concentrations were used to plot each standard accordingly, to determine unknown sugar and organic acid concentrations.

### RNA Extraction and cDNA Synthesis

Samples for RNA extraction and qPCR analysis were taken every day at the same time from two bioreactors for a period of 5 days. Volumes of 20 ml were sampled and centrifuged at 4000 rpm for 3 min. The pellet was washed in 0.9% RNAse-free NaCl and frozen in liquid nitrogen. It was thereafter stored at -80°C until extraction. RNA was extracted from the pellets as described previously (Collart and Oliviero, 1993). RNA was DNAse treated with added RiboLock RNAse Inhibitor according to protocol provided by Promega (Fitchburg, Wisconsin). Total RNA was quantified using a Nanodrop^TM^ 1000 Spectrophotometer, and the integrity of total RNA was checked on a 1.2% agarose gel both before and after DNAse treatment. RNA was of a good quality and was found to be free of genomic DNA. RNA (200 ng) was reverse transcribed to cDNA using PCR techniques according to directions supplied by the Promega ImProm-II^TM^ Reverse Transcription System kit (Fitchburg, Wisconsin).

### Gene Expression Analysis Using Quantitative Polymerase Chain PCR (qPCR)

The transcript levels of 3 ammonium transporter genes, *MEP1*, *MEP2*, and MEP3, were monitored during the co-culture period using qPCR. Yeast primers used for qPCR were designed using NCBI Primer design software and the Yeast Genome Database for *S. cerevisiae* (Table 2). Actin (*ACT1*) was used as the reference gene. All primers were checked using pure and co-culture cDNA to ensure that amplification only occurred in the relevant species (data not shown).

**Table 2:** Primers used during qPCR for amplification of *MEP1*, *MEP2*, *MEP3*, and the reference gene *ACT1* from *Saccharomyces cerevisiae*.

Gene	Function	Primer sequences
*MEP1*	Ammonium transporter (low affinity)	F: TCGACAGTTGGTCTGTGCTC
		R: TCGTGCTCTGTAGTGCCATC
*MEP2*	Ammonium transporter (high affinity)	F: GCTTGGACTATGCAGGTGGT
		R: TAAAACCACCGAGGTGACGG
*MEP3*	Ammonium transporter (low affinity)	F: TTGGGTTATGCTCCGGCATT
		R: GCTTTGTTGTGCCGTCCATT
*ACT1*	Actin (reference gene)	F: CGTGCTGTCTTCCCATCTATC
		R: CATCACCAACGTAGGAGTCTTT

Quantitative PCR was performed in total reaction volumes of 25 μl per well using polymerase chain reaction and carried out using RealQ PCR Mastermix containing SYBR Green DNA I dye, and protocols supplied were followed. Each sample was analyzed in triplicate and amplified on a real-time PCR instrument (Applied Biosystems 7500). Fold changes in RNA expression were quantified via the comparative CT method (7500 System Software, Applied Biosystems). The *MEP1*, *MEP*2, and *MEP*3 genes were normalized against the average values of *ACT1* as an endogenous control. The negative control containing no template DNA was subjected to the same procedure to exclude or detect any possible contamination.

## Results

### Isolation of Wastewater Adapted Microalgae and Yeast

Fifteen microalgae isolates and six yeast isolates were identified using 18S rRNA and ITS PCR and sequencing. Of the 15 microalgae isolates 13 were identified as *C. sorokiniana*, 1 as *Parachlorella beijerinckii* and 1 as *Meyerella plancktonica. C. sorokiniana* was cultured in the highest abundance and 4 different strains were identified after an NCBI blast search (Table 3).

**Table 3:** Microalgae isolates cultured from winery wastewater using standard microbiological culturing techniques.

Isolate	Most similar organism	Identity (%)	No. isolates
4Is3	*Chlorella sorokiniana* strain SAG 211-31	100	1
4Is 29	*Parachlorella beijerinckii*	100	1
3Is8, 3Is13, 4Is16	*Chlorella sorokiniana* strain Icheon 4	100, 99, 99	3
4Is15	*Meyerella Planktonica*	99	1
2.1, 2.3, 2.4, 2.5, 4, 5	*Chlorella sorokiniana* strain CMBB151	100	6
2.2, 2.7	*Chlorella sorokiniana* strain NZmm3W1	100	3

*Candida pseudolambica* (4 isolates) was isolated in the highest numbers followed by *Candida ethanolica* (3 isolates) and *S. cerevisiae* (2 isolates, Table 4). *S. cerevisiae* strain J2 and *Candida intermedia* strain JCM 1607 were previously isolated from winery wastewater (Malandra et al., 2003). However, different strains of these species were isolated from our winery wastewater sample. *C. sorokiniana* strain CMBB 151 and *P. beijerinckii* were selected for further co-culture studies. This was due to the fact that *C. sorokiniana* strain CMBB 151 was isolated in the highest abundance and *P. beijerinkii* has not been well researched. *S. cerevisiae* was selected as the yeast partner as it is able to ferment mannose with the release of CO_2_ which is essential for the synthetic co-culture system.

**Table 4:** Yeast isolates cultured from winery wastewater using standard microbiological culturing techniques.

Isolate	Most similar organism	Identity (%)	No. isolates
KW 1	*Candida intermedia*	100	1
KW 3	Uncultured *Saccharomycetes clone* (AWW 3)	99	1
KW 4	*Candida ethanolica*	99	3
KW 9	*Saccharomycete sp. 3AD15*	99	1
KW 13	*Candida pseudolambica* strain YHRM77	96	4
KW 15	*Saccharomyces cerevisiae* CBS 7834	100	2

### Single Culture Growth of Yeast and Microalgae

To assess physiological features, the winery wastewater isolates *C.sorokiniana* and *S. cerevisiae* were cultured as single cultures under variable temperature and pH conditions to determine optimum growth conditions. The growth media provided sufficient nutrients for the yeast and alga in the form of glucose and ammonium.

**Figure 2:**
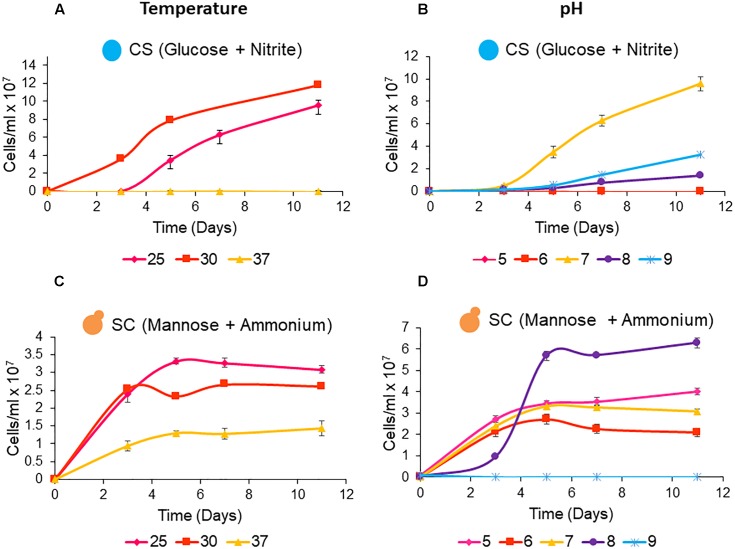
Single culture growth of microalgae and yeast under heterotrophic conditions at temperatures of 25, 30, and 37°C. **(A)** *C. sorokiniana* (CS) and **(C)** *S. cerevisiae* (SC). Single culture growth of microalgae and yeast at pH levels ranging from 5 to 9 at a temperature of 25°C. **(B)** *C. sorokiniana* (CS) and **(D)** *S. cerevisiae* (SC). All cultures were grown for 11 days from an initial inoculation density of ∼0.1 × 10^6^ cells/ml. Data represents the mean ± standard error (*n* = 3).

The data shows that *C. sorokiniana* and *S. cerevisiae* are both able to tolerate a range of growth temperatures and pH levels. The isolated strain of *C. sorokiniana* was able to grow well at temperatures of 25°C (9.6 × 10^7^ cells/ml) and 30°C (11.8 × 10^8^ cells/ml) with optimum growth observed at pH 7 (Figure 2). *S. cerevisiae* was able to grow at all tested temperatures and pH levels except for pH 9, when mannose and ammonium were supplied as carbon and nitrogen sources, respectively. Interestingly, optimal growth was observed at pH 8, which falls out of the acidophilic range normally associated with this organism (Figure 2).

### Carbon and Nitrogen Sources for Obligate Mutualistic Growth

**Figure 3:**
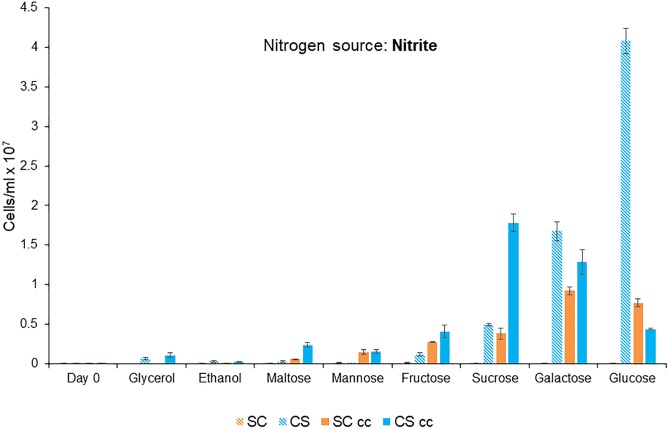
Mutualistic growth of *C. sorokiniana* (CS) and *S. cerevisiae* (SC) under single and co-culture (cc) conditions with different carbon sources. Growth experiments were conducted for 7 days at 25°C under continuous light (46 μmol m^-2^ s^-1^). The carbon source was added at a concentration of 3.6%, nitrite at 20 Mm and the cells were inoculated to a cell density of 0.1 × 10^6^ cells/ml. Data represents the mean ± SE (*n* = 3).

In the system, the microalgae partner metabolizes nitrite (NO_2_^-^) into ammonia (NH_3_^+^) which can be used as a nitrogen source by the yeast partner; while the yeast partner ferments carbon with the release of CO_2_ which can be used for microalgal growth (Table 1). The ability of *C. sorokiniana* (Figure 3) to utilize numerous carbon sources was evaluated over a period of 7 days. *C. sorokiniana* was able to utilize glucose, galactose, sucrose and fructose for growth under single culture growth conditions. *S. cerevisiae* did not grow as a single culture under any of the tested conditions as it is unable to utilize nitrite as a nitrogen source (Siverio, 2002).

Glucose was the preferred carbon source for growth with a maximum cell count of 4.1 × 10^7^ cells/ml for *C. sorokiniana* (Figure 3). *C. sorokiniana* was unable to grow in monoculture in ethanol and displayed low levels of growth when maltose (0.03 × 10^6^ cells/ml) and glycerol (0.06 × 10^6^ cells/ml) were used as carbon sources. *S. cerevisiae* did not grow in single culture under any of the conditions tested as it is unable to utilize nitrite as a nitrogen source (Siverio, 2002).

Mutualistic growth was observed for *C. sorokiniana* and *S. cerevisiae* in co-culture when maltose, mannose, fructose, sucrose, galactose, and glucose were used as carbon sources (Figure 3). *C. sorokiniana* growth was enhanced in the presence of *S. cerevisiae* when maltose, galactose, fructose, and sucrose were used as carbon sources. Growth of *C. sorokiniana* was decreased in the presence of glucose when *S. cerevisae* was present (Figure 3). Mannose was the only carbon source which induced an obligate mutualistic relationship based on co-supported growth between *C. sorokiniana* and *S. cerevisiae.*

### Co-culture Time-Course Experiments

#### Impact of Temperature on Mutualism

**Figure 4:**
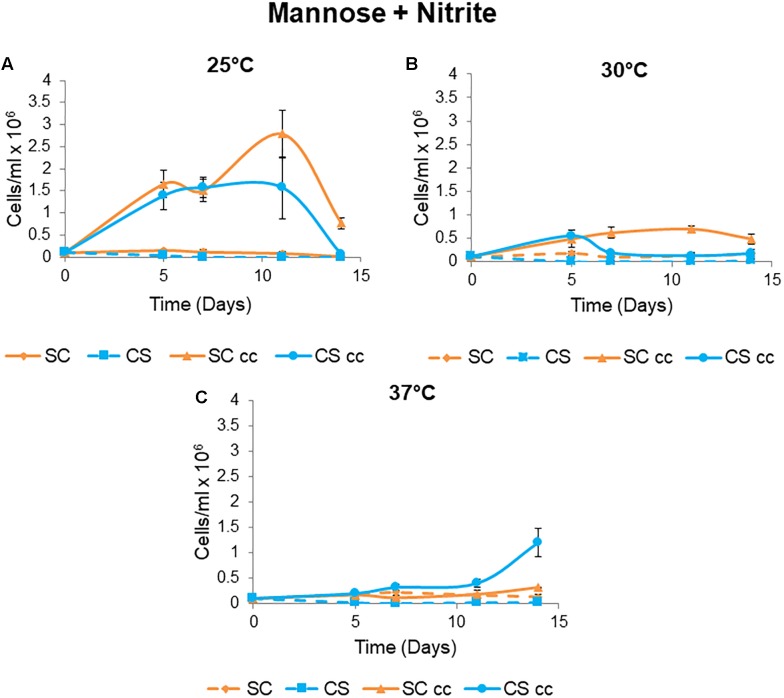
Mutualistic growth of *C. sorokiniana* (CS) and *S. cerevisiae* (SC) under single and cc conditions at different temperatures. Cultures were grown from an initial inoculum of 0.1 × 10^6^ cells/ml for each species at temperatures of **(A)** 25°C, **(B)** 30°C, and **(C)** 37°C. Data represents the mean ± standard error (*n* = 3).

To evaluate the influence of temperature on the formation of synthetic mutualisms between *C. sorokiniana* and *S. cerevisiae*, co-culture experiments were conducted at temperatures of 25 and 30°C (Figure 4). *C. sorokiniana* was unable to grow to as a single culture when *S. cerevisiae* was absent as no external CO_2_ was supplied. Similarly, *S*. *cerevisiae* single cultures were unable to grow in the absence of *C. sorokiniana* as nitrite was not metabolized to ammonia. Growth was only observed when the yeast and microalga were grown as co-cultures with *S. cerevisiae* providing *C. sorokiniana* with CO_2_ and *C. sorokiniana* providing *S. cerevisiae* with ammonia in a mutually dependent manner (Figure 4). *C. sorokiniana* and *S. cerevisiae* pairing displayed differing patterns of growth at each temperature with improved growth being observed when experiments were conducted at 25°C (Figure 4A). Growth at a temperature of 30°C was much lower than at 25°C with similar cell numbers for each species observed after 5 days, after which *C. sorokiniana* cell numbers start to decline (Figure 4B). *S. cerevisiae* can persist for longer but starts to decline after 11 days as *C. sorokiniana* is no longer able to support its growth (Figure 4B). Co-supported growth was not supported at a temperature of 37°C (Figure 4C).

#### Impact of pH on Mutualistic Growth

**Figure 5:**
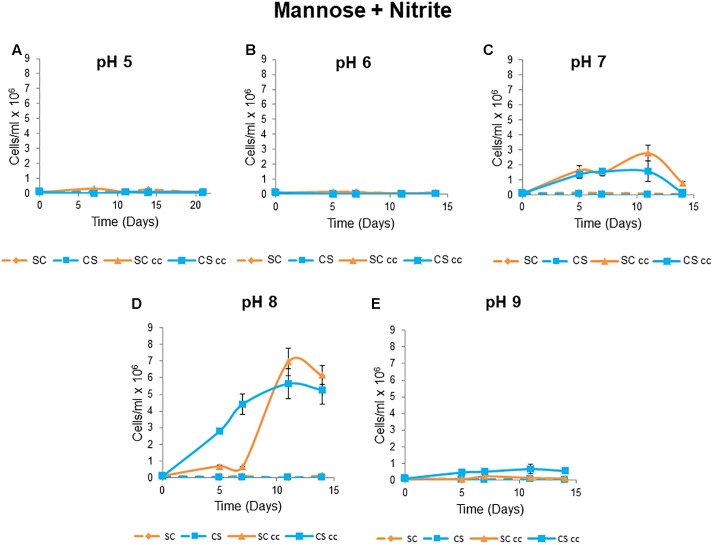
Mutualistic growth patterns of *C. sorokiniana* and *S. cerevisiae* at 5 different pH levels: **(A)** pH 5, **(B)** pH 6, **(C)** pH 7, **(D)** pH 8, and **(E)** pH 9. The growth temperature was kept constant at 25°C with no agitation under continuous light. Cells were inoculated to a cell density of 0.1 × 10^6^ cells/ml. Data represents the mean ± standard error (*n* = 3).

The co-supported growth phenotype of *C. sorokiniana* and *S. cerevisiae* were not evident at pH levels of 5 and 6 under co-culture conditions when mannose and nitrite were used as the C and N sources (Figure 5A,B). Optimal co-culture growth was observed at pH 8 with a maximum cell count of 7 × 10^6^ cells/ml for *S. cerevisiae* and 6 õ 10^6^ cell/ml for *C. sorokiniana* after 11 days (Figure 5D). Cell counts for both microorganisms increased by 3-fold at pH 8 when compared to co-culture growth at pH 7 (Figure 5C,D). At pH 9 there was no growth of the yeast and limited growth of the algae with a maximum cell count of 0.7 × 10^6^ cells/ml obtained after 11 days followed by a decline in cell numbers (0.6 × 10^6^ cells/ml) at day 14 (Figure 5E).

### Obligate Mutualistic Growth in 1L Bioreactor

**Figure 6:**
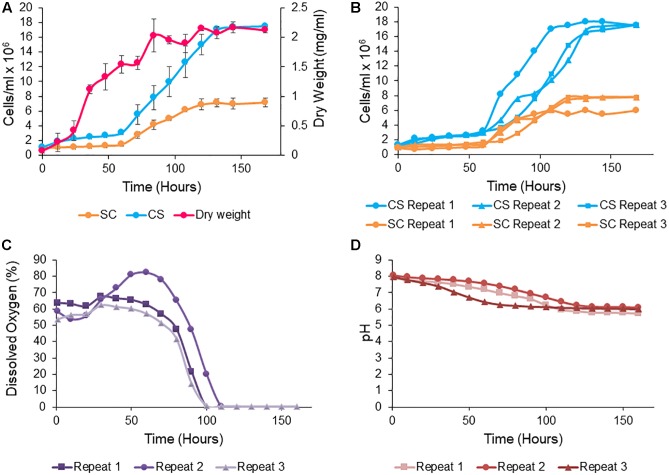
*Saccharomyces cerevisiae* (SC) and *Chlorella sorokiniana* (CS) cc cell growth (cells/ml) and combined dry weight (mg/ml) **(A)** in pH 8 TAP medium, 3.6% mannose, and 20 mM nitrite in 1 L bioreactor. Data represent the mean ± standard error (*n* = 3). **(B)** Cell density of *S. cerevisiae* and *C. sorokiniana* in 1 L bioreactor for each biological replicate. **(C)** Dissolved oxygen (%) for each biological repeat. **(D)** pH decrease measured online for each biological repeat.

Most obligate mutualism studies have been conducted on the small scale and thus the co-culture system developed here was up-scaled to the bioreactor to permit sampling of larger volumes for the monitoring of mannose, nitrite and organic acid production. *S. cerevisiae* and *C. sorokiniana* co-cultures were grown in TAP medium (pH 8) as this was the optimal growth condition for this pairing, with a cell count of 6 × 10^6^ cells/ml for *C. sorokiniana* and 7 × 10^6^ cells/ml for *S. cerevisiae* after 11 days. When up-scaled to the bioreactor, there was a significant difference between the growth of *C. sorokiniana* and *S. cerevisiae* in co-culture, with a higher cell count observed for *C. sorokiniana* (18 × 10^6^ cells/ml) compared to *S. cerevisiae* (7 × 10^6^ cells/ml) after 168 h (Figure 6A). Variation in cell numbers and dry weight was observed in the three biological repeats for both yeast and microalgae (Figure 6B), but generally the same trends were observed. All biological repeats entered exponential phase after 60 h. The combined dry weight was ∼2.125 ± 0.05 mg/ml for all biological repeats after 168 h (Figure 6A). It should be noted that while the cell numbers remain relatively constant throughout the initial culturing period, we do see an increase in the size of the cells during this period which accounts for the observed increase in the dry weight measurements. Initially the microalgae cells are quite small but as they system gets going and more nutrients are provided they do increase in size. Mannose and nitrite were consumed by *S. cerevisiae* and *C. sorokiniana*, respectively. Mannose concentrations decreased from ∼ 34 g/l to ∼ 11.6 g/l within 168 h, while nitrite decreased from an initail concentration of 0.18 g/l to 0.05 g/l. The production of organic acids, glycerol, and ethanol were measured using HPLC analysis (Table 5). There was no citric acid, tartaric acid, malic acid and acetic acid produced above the limit of quantification (LOQ). Production of succinic acid, glycerol and ethanol were observed above the LOQ in all biological repeats. Succinic acid was present with ∼ 0.121 g/l after 168 h and glycerol concentration was above LOQ in stationary phase after 132 h in all biological repeats. There was an increase in ethanol concentration observed over the LOQ after 72 h. Variation was observed between biological repeats, but succinic acid and glycerol production were detected at the same time points. However, ethanol production was consistently detected from 72 h for all biological repeats. The change in dissolved oxygen and pH were measured throughout the experiment and differences between biological repeats were observed (Figure 6C,D). Dissolved oxygen decreased with the increase in yeast cell numbers after 60 h and the pH decreased from pH 8 to ∼ 6 after 168 h in all biological repeats.

**Table 5:** Mannose and nitrite consumption and succinic acid, glycerol, and ethanol production detected above limit of quantification (LOQ) in *S. cerevisiae* and *C. sorokiniana* co-culture in 1 L bioreactor.

			Succinic
Time	Mannose^∗^	Nitrite^∗^	acid^∗^	Glycerol^∗^	Ethanol^∗^
0	33.77 ± 3.60	0.180 ± 0.03	BD	BD	BD
72	22.87 ± 1.84	0.097 ± 0.04	BD	BD	0.736 ± 0.53
96	21.86 ± 0.070	0.078 ± 0.04	BD	BD	1.349 ± 0.76
132	18.23 ± 1.17	0.063 ± 0.04	BD	0.545 ± 0.21	2.896 ± 0.38
168	11.62 ± 1.21	0.051 ± 0.04	0.109 ± 0.03	1.009 ± 0.35	5.234 ± 1.70

*Data represents mean ± standard deviation (*n* = 3). ^∗^Concentrations are given as g/l. ^∗^BD Below detection limit*.

#### Gene Expression Analysis for Batch Co-cultures

**Figure 7:**
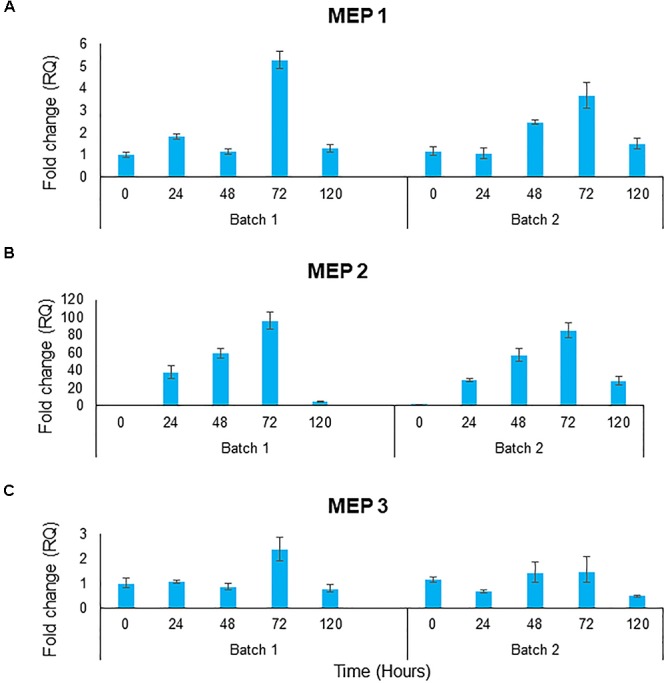
Gene expression of the ammonium transporter gene **(A)** *MEP1*, **(B)** *MEP2*, and **(C)** *MEP3* over 5 days of batch culture for 2 biological replicates. Gene expression shown in fold change of relative gene expression. Data represents 3 technical repeats for each biological repeat.

No ammonium could be measured in the medium during obligatory mutualistic growth. To indirectly assess whether ammonium was indeed released by the microalgae, the gene expression levels of the three *S. cerevisiae* ammonium transporter genes, *MEP1*, *MEP2*, and *MEP3* were assessed. These genes are tightly regulated in response to ammonium availability, with *MEP2* encoding a high affinity transporter expressed when ammonium levels are low (Marini et al., 1997). The 3 genes showed similar trends between both batch bioreactors, however, the absolute fold changes were different and consequently each set of batch data is represented individually. Ammonium transporter, *MEP1* showed the highest levels of expression on day 3, thereafter dropping to levels similar to that of day 1 (Figure 7A). *MEP2* expression shows a gradual increase over days 0 to 3 followed by a sharp decrease in gene expression on day 4 (Figure 7B). Fold changes for *MEP2* peaked just below 95 times fold change on day three. The *MEP3* gene expression levels were shown to be relatively constant (Figure 7C). Taken together, the expression data suggest that the yeast sensed low levels of ammonium, suggesting the continuous release of this compound.

#### Co-culture Growth Under Non-obligatory Conditions

**Figure 8:**
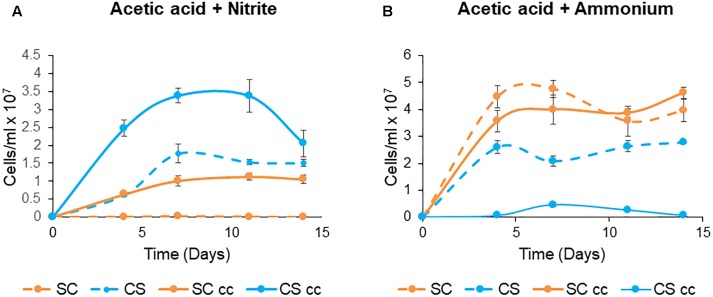
Non-obligate mutualistic growth patterns of *C. sorokiniana* (CS, **A**,**B**) with *S. cerevisiae* (SC) under single and cc growth conditions with acetic acid as the carbon source and either nitrite **(A)** or ammonium **(B)** as the nitrogen source. The growth temperature was kept constant at 25°C with no agitation under continuous light. Cells were inoculated to a cell density of 0.1 × 10^6^ cells/ml. Data represents the mean ± standard error (*n* = 3).

To evaluate whether these engineered associations provide a growth advantage to one or both species when grown in co-culture, semi-selective growth conditions were investigated. Here, acetic acid which is the carbon source normally present in TAP medium is the carbon source for the microalgae; however, the yeast is still reliant on the microalgae for the conversion of nitrite to ammonia. Under these conditions *C. sorokiniana* showed a ∼ two-fold increase in growth when co-cultured with *S. cerevisiae* compared to the single cultures (Figure 8A). Similarly, ∼ 10 fold increase in growth was observed for *S. cerevisiae* when grown with the microalgae.

In contrast, the use of ammonium as a nitrogen source created an environment in which neither organism was reliant on the other for growth and survival as both microalgae and the yeast can use ammonium as a nitrogen source. In this system, *C. sorokiniana* is rapidly outcompeted by *S. cerevisiae* under these non-obligate growth conditions (Figure 8B). Both *C. sorokiniana* and *S cerevisiae* showed improved growth when cultured as single cultures compared to co-cultures (Figure 8B).

## Discussion

Here, we have established two sets of experimental conditions for the formation of obligate and non-obligate associations between *S. cerevisiae* and *C. sorokiniana*. The initial experiments suggested that, given similar temperature and pH tolerances of these two species, stable yeast, and microalgae obligate mutualisms would be fairly easy to establish over a range of conditions. However, despite the similar temperature and pH preferences for monoculture growth of *C. sorokiniana* and *S. cerevisiae*, mutualistic growth under obligate growth conditions could not always be inferred from single culture data. This concurs with recent investigations which suggest that even a simple assemblage of two microbial genotypes can often exhibit complex and unexpected dynamics that cannot be determined from analyzing each genotype in isolation due to the wide range of positive and negative interactions which can occur (Nadell and Foster, 2012; Allen and Nowak, 2013; Dolinšek et al., 2016).

The advantages that can be derived from these co-culture systems are clear from the data presented in Figure 3. *C. sorokiniana* grew much better in co-culture when glycerol, maltose, mannose, sucrose and fructose were used as carbon sources. Similarly, the growth of *S. cerevisiae* was also improved under certain co-culture growth conditions. Often these microorganisms are grown as monocultures in the lab, however, here we see clear evidence that co-culturing these microorganisms leads to improved growth for both the yeast and microalgae under a range of different growth conditions. This is a significant result as it suggests that the interplay between different carbon and nitrogen sources is key to improving biomass production. These co-culture interactions can also have a negative impact as was observed by the suppressed growth of *C. sorokiniana* when in co-culture with *S. cerevisiae* in the presence of glucose. It is likely that under these growth conditions the microorganisms are competing for the same nutrients indicating that each microorganism might have similar nutritional requirements under these specific conditions. The nature of these of interactions require further characterization and more in-depth transcriptomic analysis is needed to provide information on how carbon and nitrogen metabolism is modulated in these co-culture systems.

The obligate nutritional dependencies established in this study were based on cross-feeding of mannose and nitrite resulting in a stable mutualism between *C. sorokiniana* and *S. cerevisiae*. The establishment of these mutualisms were strongly influenced by pH and temperature within the synthetic environment. Temperature is one of the major environmental factors governing algal growth dynamics as temperature changes have direct impacts on microalgae growth rates and membrane fluidity. Indeed, it has been suggested that changes in cytoplasmic viscosity under sub-optimal temperature conditions is responsible for less efficient carbon and nitrogen utilization (Raven and Geider, 1988; Juneja et al., 2013). Thus, there could be competitive or antagonistic interactions which are perhaps induced by temperature changes which may not facilitate mutualistic associations between *C. sorokiniana* and *S. cerevisiae* at the slightly higher temperature.

The pH tolerance strongly influenced the obligate mutualistic associations as mutualisms were unable to form at pH 5, 6, and 9 despite growth of single cultures at these pH levels. This may indicate that species-specific interactions in combination with the environmental conditions are the main drivers for the establishment of these synthetic mutualisms. This species specific effect has been demonstrated previously for algal auxotrophs and vitamin B_12_ producing heterotrophic bacteria The pH of winery wastewater can vary considerably with a range between 3 and 12 depending on the cultivar, time of year and cleaning practices (Mosse et al., 2011a; Sheridan et al., 2011; Howell et al., 2016). The fairly narrow pH range under which these obligate mutualisms are maintained at this stage indicate that this co-culture system would only be applicable to wastewater with a narrower pH range. However, it should be noted that mutualistic systems may be able to extend the survival and persistence over time of individual species. Furthermore, the conditions imposed here are highly selective, and these mutualisms may therefore be able to tolerate a wider pH range under non-obligate conditions. Of further interest is the fact that *S. cerevisiae*, an acidophilic organism, generally grows better under acidic conditions with an optimal pH ranging between 4 and 6. However, depending on temperature and the presence of oxygen, this particular strain of yeast showed improved growth at pH 8, which might be an inherent trait acquired from living in the highly variable winery wastewater environment (Narendranath and Power, 2005).

The optimized obligate mutualism in a 1 L bioreactor allowed continuous sampling without the disruption of the mutualism and decreased the risk of contamination. The system used had a higher light intensity, which was likely responsible for higher microalgal cell numbers. However, *S. cerevisiae* remained present in sufficient cell numbers to support algal growth and maintain the co-dependent growth relationship between the yeast and microalgae. Remaining mannose and nitrite after stationary phase entry and at the end of the experimental time period suggests that neither carbon nor nitrogen were the limiting factor for growth. The growth arrest may be due to the accumulation of toxic compounds or limitation in some other essential nutrients.

Of the monitored metabolites, succinic acid, which is produced during the tricarboxylic acid cycle by fermenting yeasts during the exponential and stationary growth phase, was produced during stationary phase along with glycerol and ethanol (Heerde and Radler, 1978; Arikawa et al., 1999). The oxygen produced by the microalga was rapidly respired by the yeast (Haukeli and Lie, 1976) as evidenced by the rapid decline in dissolved oxygen during the exponential growth of *S. cerevisiae.* In turn, this has a positive impact on the microalgae as oxidative stress is reduced, thus negating the inhibition of microalgae growth, and biomass production (Ugwu et al., 2007; Raso et al., 2012; Li et al., 2017). Additionally, succinic and acetic acid and other acids produced by the yeast may have contributed to the decrease in pH, even though they were under the LOQ. These acids, especially acetic acid, can also serve as additional carbon sources for the microalga (Perez-Garcia et al., 2011; Juneja et al., 2013).

In order to determine whether the yeast is assimilating ammonium from the media gene expression analysis was performed on 3 key ammonium transporter genes. The *MEP* gene family codes for transport proteins that allow ammonium to enter the cell. *MEP2* displays the highest affinity for ammonium, followed by *MEP1*, followed by *MEP3*. The *MEP* genes are subject to nitrogen control and on a good nitrogen source all three genes are repressed while on a poor nitrogen source, expression of *MEP2* is higher than *MEP1* and *MEP3* (Marini et al., 1997). The qPCR data showed results consistent with low nitrogen conditions. All three *MEP* genes showed the highest level of gene expression on day 3 of batch co-culturing. *MEP2* experienced a fold change of almost 100 times. Thus, this data provides strong evidence that ammonium is being assimilated by the yeast. As there is no useable form of nitrite in the culture media the ammonium must be produced and excreted into the media by the microalgae. Ammonium release has previously been described under certain conditions as green microalgae can excrete intracellular forms of nitrogen into the media (Peltier and Thibault, 1983; Krämer et al., 1988). This usually occurs under specific environmental conditions such as when CO_2_ levels are low and one hypothesis suggests that this might be due to a limitation of carbon skeletons for ammonia assimilation (Larsson et al., 1982).

These results also confirm that non-obligatory associations can lead to an increase in growth performance of one organism if the growth conditions are rationally modified by varying the nitrogen source (Figure 8A). This is indeed useful if the singular aim is to identify a partner which enhances biomass yields for a particular purpose or product. While nitrite enhanced the growth of the organisms in co-culture, the use of ammonium as a nitrogen source created an environment in which the microalga was rapidly outgrown by *S. cerevisiae* while still maintaining its presence at low numbers. *C. sorokiniana* grew better as a monoculture, under these non-obligatory conditions while *S. cerevisiae* showed a slight improvement in growth in co-culture. These growth experiments show that changes in an essential nutrient such as nitrogen can influence the formation of synthetic co-culture systems, driving systems either towards mutually beneficial or non-beneficial associations. This is a significant finding as most yeast-microalgae co-cultures conducted at the laboratory scale often result in the cultures being overwhelmed by the yeast and there have been relatively few studies which have focussed on optimizing these relationships (Liu et al., 2018). Here, we present a system whereby nitrogen source is used as a means of controlling the interaction between yeast and microalgae resulting in more favorable growth ratios for each microorganism in co-culture. Moreover, as both the yeast and microalgae used in this study have bioremediation capabilities in both synthetic and winery wastewater (data not shown), in the long term these co-culture systems might serve to alleviate some of the problems often associated with monoculture systems which includes low biomass yields, large input costs, and biological contamination.

The obligate mutualisms generated in this study provide the basis for the development of systems wherein ecological asscoiations between yeast and microalgae are maintained. Moreover, the autotrophic growth conditions in our obligate mutualistic system relies on a fine balance between nutrient production and nutrient utilization between the yeast and microalgae and imposes a strong selective pressure which may drive the co-evolution of these species over relatively small-time scales. We suggest that these engineered environments could be used to generate strains which display improved community traits without genetic modification. Here, we have developed a system specific for this purpose with two commercially important industrial strains. Both *S. cerevisiae* and *C. sorokiniana* are used for commercial purposes and the ability to generate both microalgae and yeast strains (GMO’s are not used in the wine industry) without genetic modification would be useful industrially. Additionally, the continued interaction might lead to strains with new metabolic traits which might survive and perform more efficiently in the harsh winery wastewater environments.

Future work will focus on the use of co-evolutionary strategies in continuous culture in combination with omics methodologies to identify the mechanisms that are involved in establishing these mutualisms. There are several questions which arise and require further investigation: Are the establishment of these mutualisms due solely to nutrient exchange or are there other metabolic or regulatory factors at play? How can we translate limited nutrient inputs into improved productivity at a minimal cost? Can we co-evolve these organisms to form co-dependent mutualistic associations independent of the selective conditions and potentially yield fitter strains? The impact of biotic factors such as the presence of bacteria should also be investigated to assess the stability and robustness of these mutualistic associations in non-sterile conditions similar to that encountered in winery wastewater environments. In conclusion, it is evident that there are numerous benefits that can be derived from these engineered co-culture assemblages when compared to monocultures. These include improved growth rates and biomass production when co-cultured as well more balanced cell ratios when appropriate nutrient sources are used. Moreover, the obligatory conditions developed here, which promote mutual dependency, could provide insight into the molecular machinery responsible for yeast/microalga ecological interactions. This could lead to the development of more robust microalga/yeast assemblages for use in a wide variety of industrial applications including biological wastewater treatment.

## Author Contributions

FB and RN conceptualized the idea, designed the experiments, and wrote the manuscript. RN, ZS, and JO performed the specific experiments. FB, RN, and ZS contributed to analysis of the experimental data. All authors were involved in revision of the manuscript and approved the final manuscript.

## Conflict of Interest Statement

The authors declare that the research was conducted in the absence of any commercial or financial relationships that could be construed as a potential conflict of interest.
